# Dipyrone use in Brazil: clinical conditions, sociodemographic characteristics, population attitudes and perceptions

**DOI:** 10.1590/0102-311XEN093125

**Published:** 2026-05-18

**Authors:** Eloá Fátima Ferreira de Medeiros, Noêmia Urruth Leão Tavares, Débora Santos Lula Barros, Tatiane da Silva Dal Pizzol, Andréia Turmina Fontanella, Dayde Lane Mendonça-Silva, Sotero Serrate Mengue, Francisco Assis Rocha Neves

**Affiliations:** 1 Programa de Pós-graduação em Ciências da Saúde, Universidade de Brasília, Brasília, Brasil.; 2 Programa de Pós-graduação em Saúde Coletiva, Universidade de Brasília, Brasília, Brasil.; 3 Departamento de Farmácia, Universidade de Brasília, Brasília, Brasil.; 4 Programa de Pós-graduação em Epidemiologia, Universidade Federal do Rio Grande do Sul, Porto Alegre, Brasil.

**Keywords:** Dipyrone, Drug Utilization, Self Report, Dipirona, Uso de Medicamentos, Autorrelato, Dipirona, Utilización de Medicamentos, Autoinforme

## Abstract

This study aims to analyze self-reported dipyrone use among the Brazilian population. We used data from the *Brazilian National Survey on Access, Use, and Promotion of Rational Use of Medicines* (PNAUM), conducted from September 2013 to February 2014 in urban areas of five regions of Brazil. The study population included individuals of all ages, with a minimum sample size of 960 interviews per sample domain, totaling 41,433 respondents and representing approximately 17 million Brazilians. We assessed socioeconomic and demographic characteristics, profiles of chronic and acute diseases, medication use, health perceptions, and perceived effectiveness and safety of dipyrone - either alone or in combination with other medications. The Poisson regression model was used to estimate adjusted prevalence ratios (aPR). The results showed that 10.4% of respondents reported using dipyrone, with 98.3% of these users taking it for acute conditions, such as pain (77.9%) and fever (14.5%). After adjusting for variables, dipyrone use for pain was more common among women (aPR = 1.66; 95%CI: 1.47-1.88) and among individuals living in the Central-West (aPR = 1.32; 95%CI: 1.04-1.67), particularly those with chronic conditions or acute events. Use for fever was similar between genders but more common among children aged under 10 years (PR = 0.62; 95%CI: 0.46-0.82). Half of the users combined dipyrone with other medications, and this practice was ten times more frequent for pain than for fever. Lastly, the effectiveness of dipyrone was widely perceived as positive, with few reports of adverse effects.

## Abstract (pt)

Este estudo tem como objetivo analisar o uso relatado de dipirona pela população brasileira. Examinamos dados da *Pesquisa Nacional de Acesso, Utilização e Promoção do Uso Racional de Medicamentos* (PNAUM), realizada entre setembro de 2013 e fevereiro de 2014, abrangendo áreas urbanas das cinco regiões do país. A população do estudo incluiu indivíduos de todas as idades, com uma amostra mínima de 960 entrevistas por domínio amostral, totalizando 41.433 respondentes e representando aproximadamente 17 milhões de brasileiros. Avaliamos características socioeconômicas e demográficas, perfil de doenças crônicas e agudas, uso de medicamentos, percepções de saúde e eficácia e segurança do uso de dipirona, isoladamente ou em combinação com outros medicamentos. A regressão de Poisson foi utilizada para estimar as razões de prevalência (RP) ajustadas. Os resultados indicaram que 10,4% dos entrevistados relataram usar dipirona, sendo que 98,3% desses usuários a utilizaram para condições agudas, principalmente dor (77,9%) e febre (14,5%). Após ajuste para variáveis, o uso de dipirona para dor foi mais comum entre mulheres (RPa = 1,66; IC95%: 1,47-1,88) e entre aquelas residentes no Centro-oeste (RPa = 1,32; IC95%: 1,04-1,67), especialmente em presença de condições crônicas ou eventos agudos. O uso para febre foi semelhante entre gêneros, porém mais comum em crianças menores de dez anos (RP = 0,62; IC95%: 0,46-0,82). Metade dos usuários combinou a dipirona com outros medicamentos, sendo essa prática dez vezes mais frequente para dor do que para febre. Por fim, a eficácia da dipirona foi amplamente percebida como positiva, com poucos relatos de efeitos adversos.

## Abstract (es)

Este estudio tiene como objetivo analizar el uso reportado de dipirona por la población brasileña. Examinamos datos de la *Encuesta Nacional de Acceso, Utilización y Promoción del Uso Racional de Medicamentos en Brasil* (PNAUM), realizada entre septiembre del 2013 y febrero del 2014, que abarcó áreas urbanas de las cinco regiones del país. La población del estudio incluyó individuos de todas las edades, con un tamaño mínimo de muestra de 960 entrevistas por dominio de muestreo, sumando un total de 41.433 encuestados y representando aproximadamente 17 millones de brasileños. Evaluamos las características socioeconómicas y demográficas, el perfil de enfermedades crónicas y agudas, el uso de medicamentos, las percepciones de salud y la eficacia y seguridad del uso de dipirona, sola o en combinación con otros medicamentos. Se utilizó la regresión de Poisson para estimar las razones de prevalencia (RP) ajustadas. Los resultados indicaron que el 10,4% de los encuestados informaron usar dipirona, y el 98,3% de estos usuarios la empleaban para afecciones agudas, principalmente dolor (77,9%) y fiebre (14,5%). Tras ajustar las variables, el uso de dipirona para el dolor fue más común entre mujeres (RPa = 1,66; IC95%: 1,47-1,88) y entre los residentes en el Centro-Oeste (RPa = 1,32; IC95%: 1,04-1,67), especialmente en presencia de condiciones crónicas o de eventos agudos. El uso para la fiebre fue similar entre géneros, pero más común en niños menores de diez años (RP = 0,62; IC95%: 0,46-0,82). La mitad de los usuarios combinó la dipirona con otros medicamentos, una práctica que fue diez veces más frecuente para el dolor que para la fiebre. Finalmente, la eficacia de la dipirona fue ampliamente percibida como positiva, con pocos informes de efectos adversos.

## Introduction

Dipyrone, also known as metamizole, is a prodrug with analgesic, antipyretic, and spasmolytic properties and low anti-inflammatory activity. It is available over the counter in countries such as China, Russia, Poland, Egypt, Brazil, and Mexico for these indications. Although concerns regarding a possible association with agranulocytosis have led to its ban in several countries, dipyrone remains widely used worldwide, particularly for treating fever and both acute and chronic pain ^1^ ^,^ ^2^ ^,^ ^3^ ^,^ ^4^.

The risk of dipyrone-related agranulocytosis, while historically debated, is considered low according to large international studies. Early concerns stemmed from its chemical similarity to aminopyrine, a compound known to carry a high agranulocytosis risk. However, recent evidence, including findings from the LATIN study ^5^, indicates a very low annual incidence in Brazil. These data support the continued therapeutic use of dipyrone when appropriately indicated ^2^ ^,^ ^3^ ^,^ ^4^ ^,^ ^6^ ^,^ ^7^ ^,^ ^8^ ^,^ ^9^ ^,^ ^10^.

Despite its long-standing clinical use, there is limited information on the profiles of individuals who use dipyrone in different settings. Previous national surveys have identified dipyrone as the most frequently used analgesic but did not explore important aspects such as whether it was prescribed by a physician or self-medication, its use in association with other drugs, or specific clinical indications ^11^ ^,^ ^12^ ^,^ ^13^ ^,^ ^14^ ^,^ ^15^ ^,^ ^16^ ^,^ ^17^ ^,^ ^18^ ^,^ ^19^.

Given its widespread utilization and the lack of detailed data on consumption patterns, this study aims to analyze self-reported dipyrone use in the Brazilian population. Specifically, it investigates (1) the frequency and prevalence according to sociodemographic and clinical characteristics; (2) ranking of dipyrone among the most frequently reported drugs; and (3) users’ perceptions regarding efficacy and related problems.

## Methods

This study used data from the *Brazilian National Survey on Access, Use, and Promotion of Rational Use of Medicines* (PNAUM, acronym in Portuguese), a population-based, cross-sectional survey conducted from September 2013 to February 2014 in urban areas across Brazil’s five geographic regions (North, Northeast, Southeast, South, and Central-West). The sample was limited to urban areas due to logistical constraints and higher costs associated with rural areas.

Sampling was probabilistic and stratified across 40 domains, defined by combinations of region, sex, and age group: (1) 0-4 years, both sexes; (2) 5-19 years, both sexes; (3) 20-39 years, female; (4) 20-39 years, male; (5) 40-59 years, female; (6) 40-59 years, male; (7) ≥ 60 years, female; (8) ≥ 60 years, male. Sampling was conducted in three stages: municipalities (primary units), census tracts (secondary units), and households (tertiary units), based on the 2010 Brazilian Census Address Registry (https://www.ibge.gov.br/en/statistics/social/population/38751-national-address-file-for-statistical-purposes.html). Municipalities were selected using systematic sampling with probability proportional to size, and households were randomly selected.

A minimum of 960 individuals per domain was required to achieve a coefficient of variation ≤ 0.05, totaling 38,400 participants. After accounting for refusals, the final sample comprised 41,433 individuals, representing the Brazilian urban population (~177 million; 93% of the national total).

In-person interviews were conducted, with data collected using standardized questionnaires installed on tablet computers. The instruments, organized into 11 thematic blocks and two medicine-specific forms, were developed and validated by researchers from seven Brazilian universities. Details and the full questionnaires are available on the PNAUM website (http://www.ufrgs.br/pnaum).

Household response rates ranged from 42% to 60%, while individual response rates varied between 82% and 97%, resulting in overall response rates ranging from 46.6% to 56.1%.

This analysis included all individuals who self-reported dipyrone (metamizole sodium) use in any section of the questionnaire addressing medication use for chronic conditions or acute events. Dipyrone use for pain and/or fever was analyzed separately.

Independent variables included: sex (male, female); age group, in years (0-10, 11-19, 20-59, ≥ 60); geographic region (North, Northeast, Southeast, South, Central-West); and socioeconomic classification (A/B, C, D/E), as defined by the Brazilian Association of Population Studies (ABEP) ^20^, based on household assets, income, and education.

Clinical variables included the number of medications in use (excluding dipyrone: 0, 1, 2, 3-4, ≥ 5), the number of chronic diseases (0, 1, 2, ≥ 3), and the number of acute events treated in the previous 15 days (0, 1, 2, ≥ 3).

Participants were asked to present all medications in current use, including industrialized and compounded products, herbal remedies, teas, and homeopathic medicines. Acute medication use was limited to the 15 days preceding the interview to reduce recall bias.

Self-rated health was assessed using the question: “Overall, how do you rate your health?” (very good, good, fair, poor, very poor). For children (< 15 years), individuals with communication difficulties, or those with cognitive impairments, information was provided by a parent or caregiver.

All medication-related data were collected using specific forms, which included drug name, pharmaceutical form, concentration, indication, prescriber (if applicable), type of use (prescribed or self-medicated), perceived effectiveness, and adverse events. Participants were asked to present prescriptions or drug packages when possible.

Medications were classified using the Brazilian Health Regulatory Agency (Anvisa, acronym in Portuguese) lists, including metamizole sodium and metamizole sodium combinations (excluding psycholeptics). Combination drugs were counted as a single item.

The ten most frequently reported medications (used alone or in combination) were ranked in descending order of frequency. Reasons for dipyrone use were categorized based on responses to predefined options for chronic conditions (e.g., hypertension, diabetes, depression) and acute conditions (e.g., infection, pain, fever, flu).

The prescribing source was assessed with the question: “Who recommended this medicine?” (physician/dentist, pharmacist, other health professional, self, family/friend/partner, pharmacy clerk, other). Self-medication was defined as drug use without a professional prescription.

Effectiveness and safety perceptions were assessed by asking whether the medication improved the condition and whether any adverse effects were experienced.

Descriptive analyses were performed using relative frequencies, with a 5% significance level. Poisson regression was used to estimate adjusted prevalence ratios (aPR), and model selection was performed using backward stepwise procedures. Variables with p ≤ 0.20 in univariate analyses were retained in the multivariate model. All analyses were performed using Stata version 13.0 (https://www.stata.com), with procedures for complex sample designs.

PNAUM was approved by the Brazilian National Commission for Research Ethics (CAAE 18947013.6.0000.0008), the highest body responsible for ethical appraisal of research protocols involving human beings in Brazil. All participants signed the informed consent form. For minors or individuals unable to provide consent, the form was signed by a parent of legal guardian.

## Results

The total sample of the population database comprised 41,433 respondents, while the medicines database included 54,166 reported medications.

Among the respondents, 4,310 individuals (10.4%; 95%CI: 9.6-11.3) reported using dipyrone, representing approximately 17.7 million Brazilians. Of these, 98.3% (95%CI: 97.6-98.8) reported using dipyrone for acute events that occurred within 15 days before the interview. Among dipyrone users, 77.9% (3,357 individuals) reported use for pain, corresponding to 8.1% of the 41,443 individuals (95%CI: 7.4-8.8); 14.5% (625 individuals) reported use for fever, corresponding to 1.5% of the total sample (95%CI: 1.3-1.8); and 7.7% (332) reported other indications.

One in three Brazilians (33.7%; 95%CI: 32.1-35.4) reported using medications for acute events in the 15 days prior to the interview. Among these users, 30.4% (95%CI: 28.8-32.0) reported using dipyrone, and over 70% indicated pain as the reason for use.

**Figure 1:**
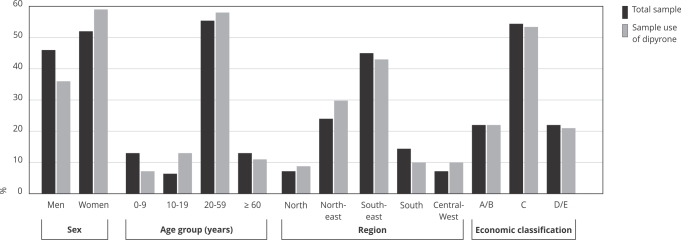
Sociodemographic characteristics of the total sample and of individuals who reported using dipyrone in Brazil (n = 41,433).

**Figure 2:**
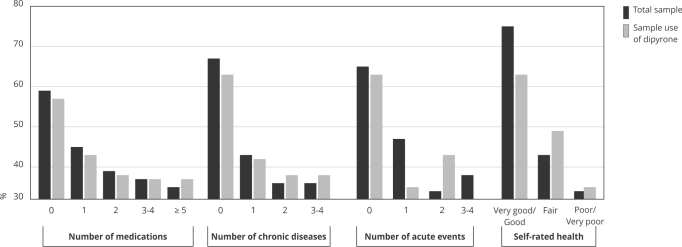
Medication use patterns and health conditions of the total sample and of individuals who reported using dipyrone in Brazil (n = 41,433).

The population reporting dipyrone use was predominantly composed of women (64.4%; 95%CI: 61.8-67.0), aged 20-59 years (67.9%; 95%CI: 65.3-70.4), residing in the Southeastern region of Brazil (43.7%; 95%CI: 37.0-50.6), and belonging to socioeconomic class C (56.8%; 95%CI: 53.9-59.6) (Figure 1). Among dipyrone users, nearly half of respondents (41.9%; 95%CI: 39.0-44.8) reported not using other medications (chronic or occasional). Most reported no chronic diseases (59.2%; 95%CI: 56.2-62.2), reported only one acute event (64.5%; 95%CI: 61.4-67.4), and rated their health as very good or good (64.4%; 95%CI: 61.3-67.0) (Figure 2).

**Figure 3:**
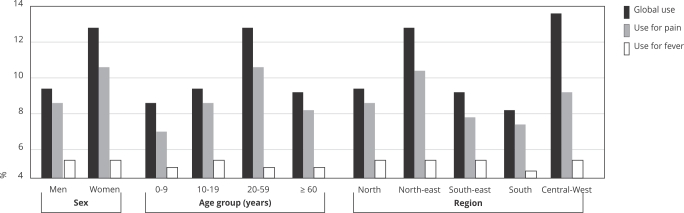
Prevalence of overall dipyrone use in the Brazilian population and prevalence of dipyrone use for pain and fever according to sociodemographic characteristics (n = 41,433).

**Figure 4:**
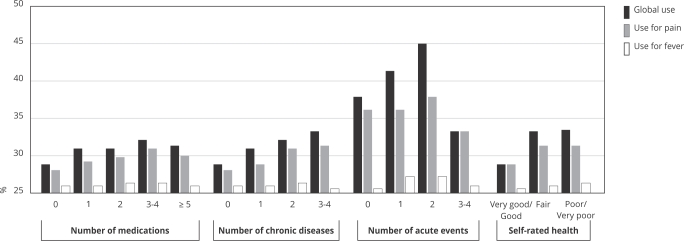
Prevalence of overall dipyrone use in the Brazilian population and prevalence of dipyrone use for pain and fever according to medication use and health condition characteristics (n = 41,433).

The prevalence of dipyrone use for pain was higher among women (10.3%; 95%CI: 9.4-11.2), individuals aged 20-59 years (10.5%; 95%CI: 9.6-11.4), and residents of the Northeast and Central-West. The prevalence of dipyrone use was higher in polypharmacy individuals (five or more medications: 12.2%; 95%CI: 10.0-14.7), those with three or more chronic diseases (13.9%; 95%CI: 11.9-16.3), those reporting three or more acute events (25.9%; 95%CI: 22.0-30.3), and those with poorer self-rated health (poor or very poor: 12.8%; 95%CI: 10.6-15.2) (Figures 3 and 4).

The prevalence of dipyrone use for fever was similar between men and women, but higher among children < 10 years old (4.4%; 95%CI: 3.5-5.4) and residents in the Central-West (2.5%; 95%CI: 1.8-3.5) (Figures 3 and 4).

After adjusted analysis (Table 1), the association with the use of dipyrone was maintained among the variable female sex in the overall population (aPR = 1.66; 95%CI: 1.47-1.87). Furthermore, considering the overall population, a negative association was observed among children < 10 years old (PR = 0.64; 95%CI: 0.52-0.77). Dipyrone use also remained positively associated with residence in the Central-West (aPR = 1.32; 95%CI: 1.04-1.67), among users with chronic diseases or acute events and poorer self-rated health. No significant associations were observed with socioeconomic class or with the use of other medications (chronic or acute).

**Table 1:** Prevalence ratio (PR) and adjusted prevalence ratio (aPR) for dipyrone use according to sociodemographic characteristics, medication use, and health conditions in the Brazilian population.

Characteristics	PR	95%CI	aPR	95%CI
Sex				
Male	Reference			
Female	1.61	1.44-1.80	1.66	1.47-1.87
Age group (years)				
0-9	0.64	0.52-0.77	NS	
10-19	1.01	0.83-1.22	NS	
20-59	1.41	1.25-1.59	0.99	0.79-1.25
≥ 60	Reference			
Region				
North	Reference			
Northeast	1.37	1.09-1.72	1.24	0.99-1.55
Southeast	1.06	0.82-1.37	1.02	0.79-1.32
South	0.76	0.59-1.00	0.78	0.60-1.00
Central-West	1.39	1.09-1.76	1.32	1.04-1.67
Economic Classification				
A/B	Reference			
C	1.03	0.89-1.20	NS	
D	0.99	0.84-1.17	NS	
E	0.84	0.62-1.13	NS	
Number of medications				
0	Reference			
1	1.42	1.24-1.64	NS	
2	1.52	1.30-1.78	NS	
3-4	1.69	1.45-1.97	NS	
≥ 5	2.01	1.71-2.36	NS	
Number of chronic diseases				
0	Reference			
1	1.32	1.16-1.50	1.10	0.97-1.26
2	1.57	1.33-1.85	1.24	1.03-1.49
3	1.92	1.60-2.30	1.53	1.26-1.87
≥ 4	1.73	1.41-2.13	1.38	1.11-1.73
Number of acute events				
0	Reference			
1	1.24	1.08-1.42	0.99	0.85-1.16
2	1.83	1.53-2.18	1.25	1.03-1.53
3	2.23	1.77-2.82	1.49	1.18-1.89
≥ 4	1.22	0.71-2.11	0.80	0.42-1.51
Self-rated health				
Very good/Good	Reference			
Fair	1.67	1.48-1.89	1.45	1.27-1.64
Poor/Very poor	1.64	1.37-1.97	1.34	1.11-1.63

95%CI: 95% confidence interal; NS: not significant.

Dipyrone was the most frequently reported medication among all active ingredients mentioned by the interviewees, representing 9.1%. Approximately half of the individuals reported using dipyrone in combination with other drugs (4.3%). In addition to dipyrone (4.8%) and dipyrone-containing combinations (4.3%), other commonly reported medications included hydrochlorothiazide (4.0%), paracetamol (3.9%), losartan (3.5%), omeprazole (3.3%), simvastatin (2.8%), metformin (2.6%), and captopril (2.5%). These figures reflect the overall distribution of the 10 most commonly reported drugs in the PNAUM study conducted in Brazil in 2014, based on the medicines atabase (54,166). Medicines whose names could not be identified were excluded. Among dipyrone-containing combinations, the most frequent were orphenadrine + caffeine (58.1%), isometheptene + caffeine (21.5%), chlorpheniramine + caffeine (8.3%), and others (4.4%).

Additionally, dipyrone was the most frequently reported medication for treating acute events, representing 20.7% (95%CI: 19.6-21.9). In more than 50% of the reports, dipyrone was associated with other active ingredients (10.5%; 95%CI: 9.7-11.3).

Subsequently, the attitudes and perceptions of individuals who used dipyrone alone or combined with other medications were compared. For pain treatment, combined use of dipyrone was more frequently reported (84.1%; 95%CI: 81.3-86.5) than isolated use (69.7%; 95%CI: 67.6-71.7). Conversely, dipyrone was more often used alone for fever (24.9%; 95%CI: 21.8-28.3) than in combination with other drugs (2.3%; 95%CI: 1.2-4.2). Prescription-based use of dipyrone was more prevalent when it was used alone (34.4%; 95%CI: 31.8-37.2), while self-medication was primarily associated with combined formulations (82.5%; 95%CI: 79.5-85.1), although it was also common with isolated use (59.6%; 95%CI: 56.7-62.5). Medical prescription for combined dipyrone formulations was relatively uncommon (13.9%; 95%CI: 11.6-16.6). Perceived effectiveness was similarly high in both groups (88% for isolated use and 87.1% for combination use), while perceived safety concerns were minimal in both (3.8% and 3%, respectively). These findings suggest that whether dipyrone was used alone or in combination did not significantly influence users’ perception of its effectiveness or safety.

## Discussion

This study presents, for the first time, the profile of self-reported dipyrone use among the Brazilian urban population. It identifies population characteristics, regional prevalence, and perceptions of its effectiveness and safety. One in ten Brazilians uses dipyrone, mainly for acute events such as pain and fever, consistent with findings from smaller studies conducted in specific regions or age groups in Brazil ^21^ ^,^ ^22^ ^,^ ^23^ ^,^ ^24^ ^,^ ^25^ ^,^ ^26^ ^,^ ^27^ ^,^ ^28^ ^,^ ^29^.

Dipyrone regulation varies globally: while it is banned in some countries, it is considered a first-line non-opioid analgesic in others, such as Germany, Serbia, and Switzerland. In Germany, where a prescription is required, outpatient dipyrone use increased fifteenfold between 1991 and 2018. In Brazil, it has been sold over the counter since the 1920s ^25^ ^,^ ^27^ ^,^ ^28^ ^,^ ^29^ ^,^ ^30^ ^,^ ^31^ ^,^ ^32^ ^,^ ^33^ ^,^ ^34^ ^,^ ^35^ ^,^ ^36^ ^,^ ^37^ ^,^ ^38^ ^,^ ^39^ ^,^ ^40^.

Our results show widespread dipyrone use across all age groups and regions, particularly for flu-like syndromes and acute pain conditions such as headache, backache, dysmenorrhea, and gastrointestinal discomfort. Dipyrone’s well-established analgesic and antipyretic properties justify its use for these conditions ^10^ ^,^ ^41^ ^,^ ^42^ ^,^ ^43^.

Higher dipyrone use was observed among young adults (20-59 years), women, individuals residing in the Northeast and Central-West, those exposed to polypharmacy, people with multimorbidity, and individuals reporting poor self-perceived health. Women tend to use medications more frequently, which is often attributed to greater health-seeking behavior and higher prevalence of painful conditions, including dysmenorrhea and headache ^1^ ^,^ ^2^ ^,^ ^7^ ^,^ ^44^ ^,^ ^45^.

Dipyrone use was higher in young adults than in children or older adults, likely reflecting a higher occurrence of acute pain events and fewer chronic conditions. In contrast, in countries such as Germany, Serbia, and Switzerland, dipyrone is more commonly used by older adults for chronic pain ^22^ ^,^ ^25^ ^,^ ^33^ ^,^ ^39^ ^,^ ^46^ ^,^ ^47^.

The increased use in Brazil’s Northeast and Central-West may be associated with increased self-medication practices, potentially due to reduced healthcare access. Self-medication with dipyrone was especially frequent for pain, requiring further investigation into regional disparities ^7^ ^,^ ^15^ ^,^ ^29^.

Although not the primary focus of this study, polypharmacy and multimorbidity likely increase analgesic use, given the higher prevalence of pain-related symptoms and adverse drug reactions such as headache and abdominal pain ^21^ ^,^ ^33^.

For fever, dipyrone use was more common among children < 10 years old, with no differences observed between sexes. Fever in children often results from respiratory infections, explaining the prevalent use of dipyrone as an antipyretic. This differs from countries such as Germany and the Netherlands, where dipyrone is rarely used as an antipyretic in children. Previous Brazilian studies also report frequent dipyrone use among children, although often many do not specify the clinical indication. Importantly, the risk of agranulocytosis in children lacks robust evidence ^6^ ^,^ ^11^ ^,^ ^26^ ^,^ ^30^ ^,^ ^48^.

Dipyrone is among the most widely used drugs in Brazil and is often considered a first-line option in primary care for various painful conditions. It is also extensively used to treat fever, especially in children. In our study, dipyrone, alone or in combination, was the most frequently reported medication. This may be due to its low cost and over-the-counter availability in community pharmacies. The most commercially successful formulation is a combination of orphenadrine and caffeine, with annual sales exceeding 500,000 units ^4^ ^,^ ^21^ ^,^ ^22^ ^,^ ^26^ ^,^ ^29^.

Dipyrone monotherapy was more frequently used for fever, while combinations were common for pain, possibly because Brazil lacks fever-targeted combination drugs. Pain management often involves multimodal analgesia using drugs with different mechanisms. However, no current evidence supports the superior efficacy of dipyrone combined with muscle relaxants or caffeine ^22^ ^,^ ^26^ ^,^ ^29^.

Most users reported self-medication, likely due to easy access and perceived effectiveness. Similar trends have been observed among adolescents, adults, older adults, and international populations. While self-medication may reduce pressure on health services, it is typically limited to minor conditions ^2^ ^,^ ^7^ ^,^ ^21^ ^,^ ^22^ ^,^ ^23^.

Interestingly, self-medicated dipyrone use was more common in combination forms, while medical prescriptions more often favored monotherapy, possibly to minimize adverse effects and enable better dose control ^2^ ^,^ ^3^ ^,^ ^4^ ^,^ ^21^ ^,^ ^22^ ^,^ ^36^ ^,^ ^37^ ^,^ ^38^ ^,^ ^39^ ^,^ ^40^ ^,^ ^46^.

Eight out of ten users perceived dipyrone as effective, and only 3% reported discomfort. A related study conducted in the same population found no serious adverse events linked to dipyrone. Reported adverse effects - such as rash or pruritus - were typically mild and resolved after drug discontinuation. Overall, existing evidence supports dipyrone’s favorable safety profile compared to other analgesics ^1^ ^,^ ^2^ ^,^ ^7^ ^,^ ^33^.

Although the prevalence of agranulocytosis does not limit the marketing of dipyrone in some countries, this adverse outcome may be strongly influenced by the concomitant use of other medications. In particular, combining dipyrone with myelotoxic or enzyme-inducing drugs may potentiate hematological toxicity or alter its metabolism, thereby increasing the risk of agranulocytosis and other adverse events. Notably, over-the-counter availability in Brazil does not imply the absence of risk. On the contrary, easy access may favor self-medication and unsupervised polypharmacy, underscoring the need for active monitoring by healthcare professionals of dipyrone use in combination with other agents known to induce agranulocytosis, aiming to minimize preventable adverse events ^2^ ^,^ ^3^ ^,^ ^8^ ^,^ ^10^ ^,^ ^33^ ^,^ ^40^ ^,^ ^41^ ^,^ ^42^ ^,^ ^43^.

This study has some limitations. Data collected from separate respondent and medication databases, hindering direct association analysis. Moreover, all information was self-reported, with potential recall bias, especially for medications used in acute conditions. Seasonal variations in illness patterns may also have influenced the prevalence of dipyrone use.

Despite these limitations, this study represents the most comprehensive population-based survey on dipyrone use in Brazil to date. It included 41,433 individuals from all five regions, representing an estimated 177 million people, and provides an original and robust overview of dipyrone use in the country.

## Conclusion

Dipyrone was the most frequently reported active ingredient among Brazilians and was predominantly used for self-medication. Approximately 10% of the Brazilian population reported dipyrone use, mainly for acute clinical conditions, especially pain, with higher prevalence among young adults. It was more common in children < 10 years of age to use this medication for fever. From a therapeutic perspective, dipyrone was perceived as highly effective, with minimal reports of adverse effects. Nevertheless, further studies are necessary to better assess the safety of dipyrone across different clinical conditions within the Brazilian population.

## Data Availability

Not applicable.
